# The Orphan G Protein-Coupled Receptor Gene GPR178 Is Evolutionary Conserved and Altered in Response to Acute Changes in Food Intake

**DOI:** 10.1371/journal.pone.0122061

**Published:** 2015-06-05

**Authors:** Vanni Caruso, Madeleine Le Grevés, Shahrzad Shirazi Fard, Tatjana Haitina, Pawel K. Olszewski, Johan Alsiö, Helgi B. Schiöth, Robert Fredriksson

**Affiliations:** 1 Department of Neuroscience, Unit of Functional Pharmacology, Uppsala University, Uppsala, Sweden; 2 Department of Biological Sciences, University of Waikato, Hamilton, New Zealand; CRCHUM-Montreal Diabetes Research Center, CANADA

## Abstract

G protein-coupled receptors (GPCRs) are a class of integral membrane proteins mediating physiological functions fundamental for survival, including energy homeostasis. A few years ago, an amino acid sequence of a novel GPCR gene was identified and named GPR178. In this study, we provide new insights regarding the biological significance of Gpr178 protein, investigating its evolutionary history and tissue distribution as well as examining the relationship between its expression level and feeding status. Our phylogenetic analysis indicated that GPR178 is highly conserved among all animal species investigated, and that GPR178 is not a member of a protein family. Real-time PCR and *in situ* hybridization revealed wide expression of Gpr178 mRNA in both the brain and periphery, with high expression density in the hypothalamus and brainstem, areas involved in the regulation of food intake. Hence, changes in receptor expression were assessed following several feeding paradigms including starvation and overfeeding. Short-term starvation (12–48h) or food restriction resulted in upregulation of Gpr178 mRNA expression in the brainstem, hypothalamus and prefrontal cortex. Conversely, short-term (48h) exposure to sucrose or Intralipid solutions downregulated Gpr178 mRNA in the brainstem; long-term exposure (10 days) to a palatable high-fat and high-sugar diet resulted in a downregulation of Gpr178 in the amygdala but not in the hypothalamus. Our results indicate that hypothalamic Gpr178 gene expression is altered during acute exposure to starvation or acute exposure to palatable food. Changes in gene expression following palatable diet consumption suggest a possible involvement of Gpr178 in the complex mechanisms of feeding reward.

## Introduction

G protein-coupled receptors (GPCRs) are a class of integral membrane proteins mediating endogenous signals from the outside of the cell into cellular responses, through activation of GTP-binding proteins (G-proteins). GPCRs play fundamental biological roles in the maintenance of vital functions, including embryonic development, blood pressure and food intake sensing signalling molecules, such as neurotransmitters and hormones [1, 2]. Alterations in the physiological status of GPCRs are often associated with underlying causes of disease [2, 3]. Hence, GPCRs are the most pursued targets for drug development constituting the target of one-third of all marketed drugs [2].

Based on sequence similarity within their transmembrane regions, mammalian GPCRs are divided into five subfamilies named *Rhodopsin* (class A or 1), *Glutamate* (also termed class C or 3), *Adhesion*, *Frizzled* and *Secretin* (class B or 2), of which *Rhodopsin* are the largest group consisting of more than 670 human GPCRs including olfactory receptors [3, 4]. The *Glutamate* family includes eight metabotropic glutamate receptors, two heterodimeric gamma-aminobutyric acid (GABA) receptors, a calcium-sensing receptor, three taste receptors, a promiscuous L-alpha-amino acid receptor (GPRC6A), and five orphan receptors [5]. Adhesion receptors, grouped initially with class B Secretins, have a very distinct GPCR proteolytic (GPS) domain, which acts as an intercellular autocatalytic site yielding two non-covalently attached subunits [6]. This family is ancestral to the Secretin family having a hormone binding domain in the N-terminus together with residues at the outer surface of the TM regions and forming a binding pocket for the ligand [7]. Frizzled receptors are a family of 11 human receptors playing a crucial functional role in both normal development and in disease sensing Wnt signalling pathways [8].

While many GPCRs have received large attention, there are several orphan GPCRs that still have obscure functions [9]. Searches with the Hidden Markov Models in the Human Genscan dataset allowed us to identify a new human GPCR, which was named GPR178 [4]. In this study, we provide novel insights regarding the functional implication of the GPR178 gene in the control of energy homeostasis using phylogenetic analysis, gene profile expression in both mouse and rat tissues, as well as several mouse and rat feeding paradigms.

## Material and Methods

All animal work was approved by the Animal Care and Ethics Committee of Uppsala, Sweden and followed the guidelines of European Communities Council Directive (86/609/EEC).

### Phylogenetic analysis

#### Recovery of protein sequences

**Fig 1:**
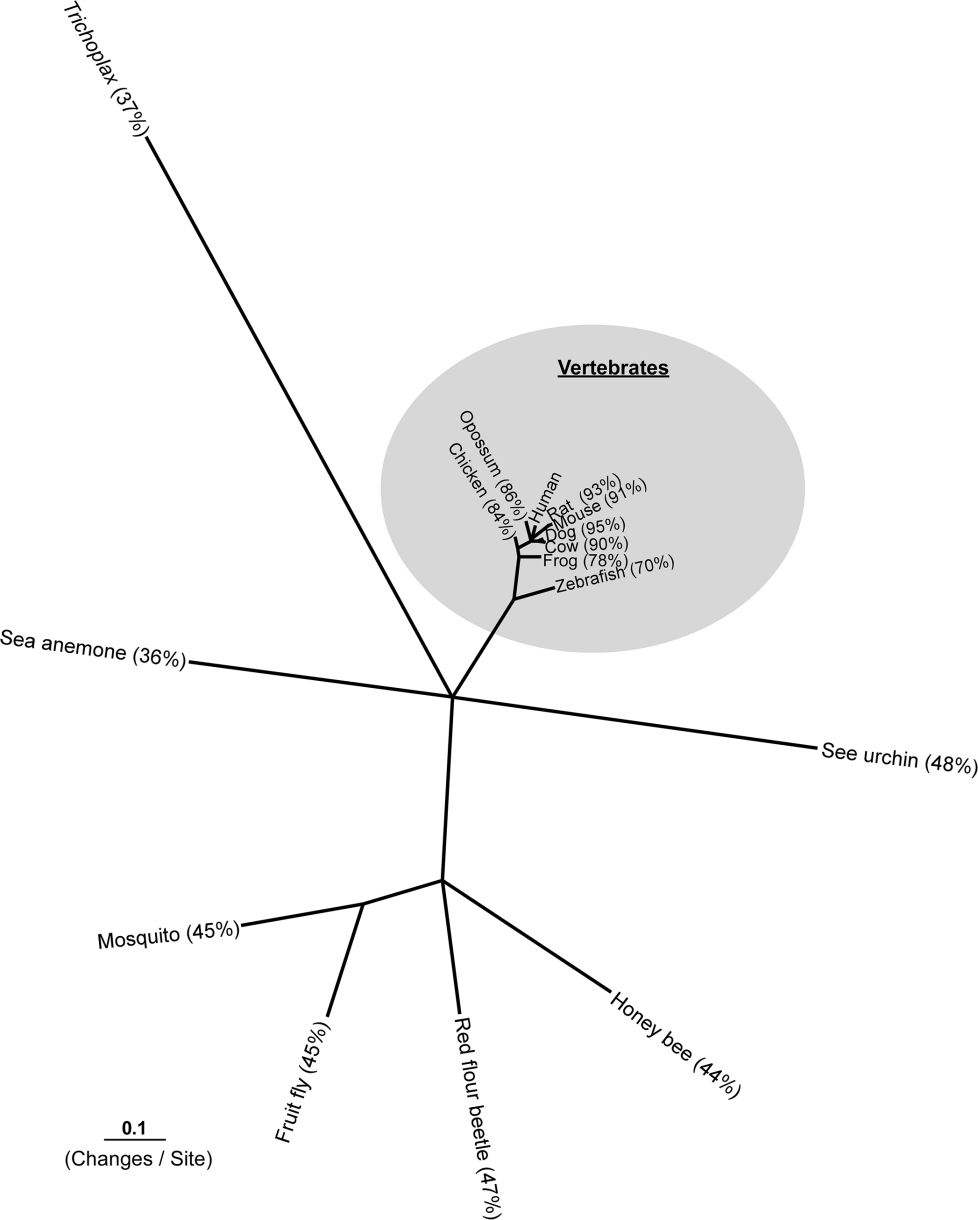
Consensus phylogenetic tree of GPR178. The consensus phylogenetic tree of GPR178 amino acid sequences from 16 different species. The numbers in percentage indicates the amino acid identity to human GPR178. The sequence alignment used for phylogenetic calculations was based on sequences starting from TM1 and constructed with MAFFT-GINSI. The consensus tree is based on 100 Maximum Parsimony trees and the branch lengths of the tree are calculated with Tree-Puzzle 5.2 using JTT model of substitution on the topology obtained from parsimony. Alignment and phylogenetic analysis of GPR178 amino acid sequences: GPR178 protein sequences were retrieved from GenBank using tblastn (http://blast.ncbi.nlm.nih.gov/Blast.cgi): human (Homo sapiens) NP 065874, house mouse (Mus musculus) NP 001028350, Norway rat (Rattus norvegicus) NP 001124411, domestic cow (Bos taurus) XP 870842, dog (Canis lupus familiaris) XP 855028, gray short-tailed opossum (Monodelphis domestica) XP 001381390, chicken (Gallus gallus) XP 419698, western clawed frog (Xenopus tropicalis) NP 001039119, zebrafish (Danio rerio) NP 001038305, purple sea urchin (Strongylocentrotus purpuratus) XP 001183797, fruit fly (Drosophila melanogaster) NP 651004, malaria mosquito (Anopheles gambiae) XP 311316, honey bee (Apis mellifera) XP 624574, red flour beetle (Tribolium castaneum) XP 969527, starlet sea anemone (Nematostella vectensis) XP 001618510 and Trichoplax adhaerens XP 002118380.

Orthologous proteins from the 16 species were investigated using Translated BLAST (tblastn) from the NCBI Non-redundant database [10]. Sequences were aligned with ClustalW 1.83 and the alignment was used to construct the HMM model using the HMMbuild and HMMpress from the HMMER package. The HMMscan identified the most similar proteins in the genomes of the 16 species [11] (Table 1). The transmembrane (TM) segments were predicted with Phobius (http://phobius.cbr.su.se/cgi-bin/predict.pl). The amino acid sequences excluding N-termini were aligned with MAFFT-GINSI (http://align.bmr.kyushu-u.ac.jp/mafft/online/server/). The alignment was bootstrapped 100 times using SEQBOOT and 100 Maximum Parsimony trees were calculated with PROTPARS from Phylip 3.67 (http://evolution.genetics.washington.edu/phylip.html). The consensus tree was calculated and used together with alignment as input files for calculations of branch lengths with Tree-Puzzle 5.2. (http://www.tree-puzzle.de/). Branch lengths were calculated with 10 000 puzzling steps using JTT model of substitution and a mixed model of heterogeneity with 1 invariable and 8 Gamma rates [12]. The output tree was plotted with Treeview 1.6.6 (http://taxonomy.zoology.gla.ac.uk/rod/treeview.html) and manually edited in CANVAS (Fig 1).

**Table 1:** Similarity of GPR178 to other proteins in the 16 species investigated.

Specie	Best hit (Accession; P value)	Second best hit (Accession; P value)
Human	IGFL2 (ENSP00000395219; 3.4E-0.05)	GPR3 (ENSP00000363136, 9.8E-5)
Mouse	SLC41A43(ENSMUST00000044019; 2E-5)	TMEM181B (ENSMUSG00000079729; 1.5E-89)
Chicken	HDAC8 (ENSGALP00000007696;1.2E-4)	*Rho GTPase activating protein 20* (ENSGALP00000007456; 1.6E-5)
Fugu	*RhoGEF* (ENSTRUT00000043577; 1.4E-)	*DNA polymerase III* (ENSTRUP00000039654; 9.8E-5)
Ciona	*RNA Exonucleas 4* (ENSCINP00000021261; 6.1E-6)	*sorting nexin-30* (ENSCINP00000011281; 3.2E-5)
Drosophila	G protein s alpha60 (FBgn0001123; 2.8E-5)	Transcription factor FREAC-2 (FBgn0050154; 4E-5)
C. elegans	7TM chemoreceptor srw-61 (H25K10.7; 5.9 E-6)	7TM chemoreceptor srx-129 (F55B12.9; 1.1E-5)
Nematostella	*RNA exonuclease 4* (jgi|Nemve1|43493; 1.7e-5)	*mitochondrial ribonuclease P protein 3 precursor* (jgi|Nemve1|233837|; 8.6E-5)
Trichoplax	*prolyl 4-hydroxylase subunit alpha-1 isoform 2 precursor* (jgi|Triad1|59907; 4.7E-5)	*protein ALEX isoform g* (jgi|Triad1|35829|; 1.1E-5)
Yeast	*No hit* (YGR064W; 1.3E-5)	*No hit* (YOR169C; 8.4E-5)
Dictyostelium	*No hit* (DDB0302642; 2.6E-5)	*No hit* (DDB0188684; 7.9E-5)
Arabidopsis	F-box protein (AT3G27290; 4.2E-6)	terpene synthase/cyclase family protein (AT4G20230; 1.9E-5)

E-values are based on blastp searches in the nr database, with output limited to the specific specie. Protein names in italic font indicates that these proteins are not annotated in the database and that we have obtained these annotations by blast searches against the human genome and used the best hit as annotation.

### Animal handling procedures and sampling

**Fig 2:**
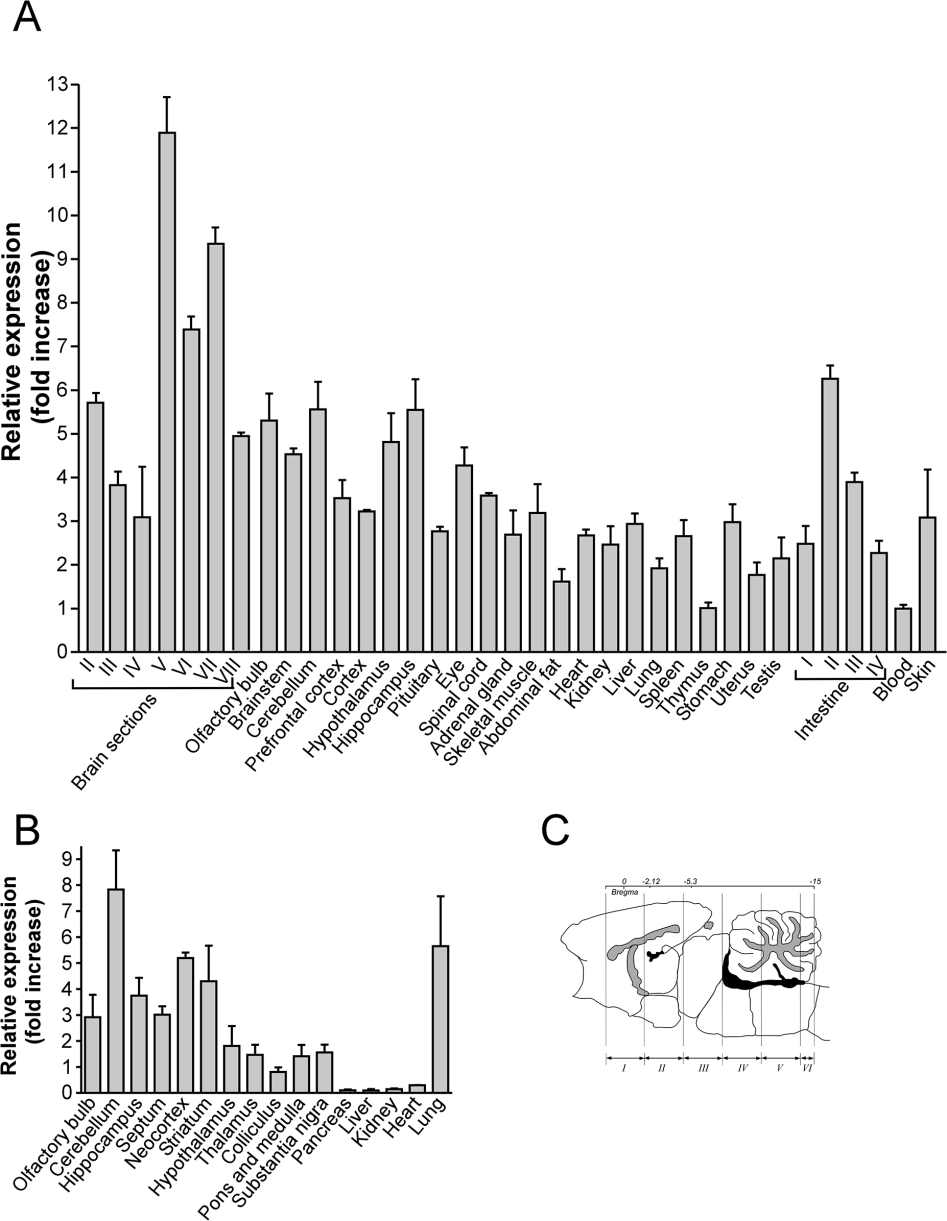
Gpr178 mRNA expression in rat and mouse. mRNA expression levels of Gpr178 in tissues from rat (A) and mouse (B). Results are shown as relative expression to minimum (fold increase). In rat panel (A), abbreviations II–VIII indicate brain cross sections adapted from [13]. Intestine was divided into four parts of equal size during dissection and the most proximal 25% denoted I and the most distal 25% denoted IV.

Rats and mice were bought from Scanbur BK AB (Sollentuna, Sweden) and Taconic M&B A/S (Ejby, Denmark), respectively. Animals were housed in standard macrolon cages with aspen wood bedding (Scanbur AB Sollentuna, Sweden) and a wooden house (rats/mice) or a paper shelter (mice). Temperature was maintained at 21–22°C, humidity 45–55% and a 12-h light/12-h dark cycle was used. Animals of similar age (7 to 9 weeks old) were used for our experiments on food intake. Rodents were handled once or twice daily and had free access to standard food pellets (R36, Lactamin, Labfor, Stockholm, Sweden) and water until treatments or behavioral studies. Brains from Sprague-Dawley rats and C57BL/6 mice were either dissected into eight coronal sections, ~3 mm thick using a brain matrix (Pelco International, Canada), or into specific brain structures (Fig 2). In addition, several organs and peripheral tissues from both rats and mice were included in the panel (Fig 2). After dissection, tissues were immediately transferred and stored in the RNAlater solution (Ambion, Austin, TX, USA) at room temperature for 1h, and then at -80°C for further investigations.

### Food intake experiments

#### Gpr178 gene expression following acute food deprivation in mice

Twenty-four male C57BL/6J mice (Taconic M&B, Denmark) of similar age and body weight, were divided into three groups of 8 mice. The control group had free access to food pellets (Lactamin, Labfor, Stockholm, Sweden) during the 24-hour experiment, while the 12-h deprived and 24-h deprived groups had no access to food for 12 and 24 hours respectively. At the end of the food deprivation period, mice were decapitated after cervical dislocation, and brains were rapidly removed. Whole hypothalamus was dissected and frozen in liquid nitrogen and stored at −80°C for subsequent RNA extraction.

#### Gpr178 gene expression following chronic food restriction and acute food deprivation in rats

Twenty-four outbred male Sprague-Dawley rats (SDsca, Scanbur BK AB, Sollentuna Sweden) of similar body weight, were divided into three groups named control, restricted and food-deprived. Control group had free access to food, whereas the restricted group was given 45% of the total food consumption compared to control group. Deprived group had free access to food until 48h before the end point of the experiment. The experiment continued for 12 days and bodyweight of rats was monitored every four days. During this period, control animals gained 40±1.5% in body weight, whereas the food-restricted animals lost 8±0.9%. At the endpoint, rats were anesthetized (pentobarbital; 60 mg/ml 50 ml APL, Sweden) and decapitated. Whole hypothalamus and prefrontal cortex were dissected, frozen in liquid nitrogen and stored at −80°C for subsequent RNA extraction.

#### Gpr178 gene expression following chronic food restriction in rats

Sixteen outbred male Sprague-Dawley (SDsca, Scanbur BK AB, Sollentuna Sweden) of similar body weight, were randomized into two groups named control and food-restricted. Rats were housed in pairs. Control group was fed *ad libitum*, while the food-restricted group was pair-fed to 40–50% of the food consumed by control group. During the 12 days experiment, food consumption and bodyweight were registered daily. At the endpoint, rats were anesthetized (pentobarbital; 60 mg/ml 50 ml APL, Sweden) and then decapitated. Amygdala was dissected, frozen in liquid nitrogen and stored at −80°C for subsequent RNA extraction.

#### Gpr178 gene expression following high fat and sugar diets in mice

Twenty-four male C57BL/6J mice, 12 weeks old and body weight average of 28g, were equally divided into three experimental groups named chow, intralipid and sucrose. Animals were housed in pairs and were given chow (R36, energy content: 3.6kcal/g, Lactamin, Labfor, Stockholm, Sweden), Intralipid (4.1% v/v solution, energy content: 0.4kcal/g, Fresenius, Sweden) or sucrose (10% w/v solution, energy content: 0.4kcal/g) for 48 hours. Food quantity and energy intake was measured. Total caloric intake per mouse was 10.3 kcal in the chow group, 14.1 kcal in the sucrose group, and 12.9 kcal in the Intralipid group. Mice were decapitated after cervical dislocation and brains were rapidly removed after decapitation. Whole hypothalamus was dissected and frozen in liquid nitrogen and stored at −80°C for subsequent RNA extraction.

#### Gpr178 gene expression following diet-induced overweight in mice

Pair-housed male C57BL/6J mice of the same initial weight (controls: 27.6 ± 0.3 g; sucrose: 27.8 ± 0.5 g) had free access to standard chow (R36, Lactamin, Labfor, Stockholm, Sweden) and water *ad libitum*. In addition, half of the animals had free access to 10% sucrose solution, *w/v*, during 3 weeks. After 3 weeks of chow and sucrose intake, mice weighed 32.1 ± 0.4 g, whereas the chow controls weighed 29.5 ± 0.6 g (P<0.05; t-test). Mice were decapitated after cervical dislocation and the whole hypothalamus was dissected and frozen in liquid nitrogen, and stored at −80°C for subsequent RNA extraction.

#### Gpr178 gene expression following schedule feeding with sweet-tasting food in rats

Twenty adult male Sprague–Dawley rats (SDsca, Scanbur BK AB, Sollentuna Sweden) had access to food 1h per day (10:00–11:00), during 14 days of experiment. Half of the animals were fed regular rodent chow (R36, Lactamin, Labfor, Stockholm, Sweden), and the other half was fed sweet-tasting rat chow (Bio-serv, F0021, Frenchtown, NJ). Food intake and body weight was recorded. Rats were decapitated at day 14 and the whole hypothalamus was dissected and frozen in liquid nitrogen, and stored at −80°C for subsequent RNA extraction.

#### Gpr178 gene expression following high-fat, high-carbohydrate preference in rats

Outbred male Wistar rats (Scanbur BK AB, Sollentuna, Sweden) were housed individually and food intake was measured twice a day. To generate a baseline, rats were given free access to regular pellets (R36, Lactamin, Labfor, Stockholm, Sweden) and water for the first three days and energy intake was measured. After that, in addition to chow, they were given either 30% sucrose solution w/v (n = 10), 100% lard (n = 10) (Dragsbæk, Denmark) or both (n = 15) for 10 days. Animals increased their caloric intake when presented with palatable diets (229 ± 3.4 kcal/kg on chow and 276 ± 5.7 kcal/kg on palatable diets; P < 0.001), but body weight did not differ between groups. At the end of experimental period, rats were decapitated and hypothalamic ARC and PVN, as well as amygdala were dissected and frozen in liquid nitrogen, and stored at −80°C for subsequent RNA extraction.

### Quantitative real-time PCR

Selected tissues were homogenized by sonication (Branson sonifier, USA) in the TRIzol reagent (Invitrogen, Sweden). Total RNA was extracted by adding chloroform and centrifuged at 10,000 x g at 4°C for 15 min. The water phase was collected and total RNA was precipitated with isopropanol after centrifugation. RNA pellets were washed in 75% EtOH, air-tried and resolved in DEPC-treated water. Samples were treated with DNase I (Roche Diagnostics Scandinavia AB, Bromma, Sweden) to remove genomic DNA for 4h at 37°C. RNA concentration was determined using a NanoDrop ND-1000 Spectrophotometer (NanoDrop Technologies, Wilmington, DE, USA). RT reaction was performed using M-MLV reverse transcriptase (GE Healthcare, Sweden) and random hexamers as primers (d(N)_6_, GE healthcare, Sweden) according to the manufacturer’s description. Total RNA (5μg), 0.25 μg primer, and 200U reversed transcriptase, 10 mM DTT, 0.5 mM dNTP in total volume of 20 μl were incubated at 37°C for 1h. Primer sequences used in the RT-PCR were designed using Beacon design v. 7.5 (Premier Biosoft, Palo Alto, CA, USA) and all primer pairs were individually evaluated for the mouse and rat, respectively. Seven housekeeping genes, including ACT (β-actin), CYCLO (cyclophilin), GAPDH (glyceraldehydes-3-phosphate-dehydrogenase), H3 (histone 3), RPL19 (ribosomal protein L19), SDCA (succinate dehydrpgenase complex, subunit A) and TUB (β-tubulin) were used to generate a normalization factor for relative expression levels in rat and mouse tissues (Table 2). The amplification was carried out in a total volume of 20 μl consisting of cDNA equivalent to 25 ng RNA, 0.25μM of forward and reverse primer, 20 μM Tris-HCl (pH: 8.4), 50 μM KCl, 4 μM MgCl_2_, 0.2 μM dNTP, SYBR Green (1:50 000) and 0.02 U/μl Taq DNA polymerase (Invitrogen, Sweden). PCR reactions were prepared in duplicates and heated to 95°C for 3 min followed by 50 cycles of denaturation at 95°C for 15 seconds (s), at optimal annealing temperature for 15 s, and extension at 72°C for 30 s. Negative and positive controls as well as a melting curve were included in each run to validate the method. RT-PCR raw data were analyzed using the MyiQ software v. 1.04 (Bio-Rad Laboratories AB, Sundbyberg, Sweden). Primer efficiencies was evaluated using LinRegPCR program (Ramakers et al. 2003), and relative expression levels using the GeNorm software [14].

**Table 2:** Primers used in real-time PCR.

Genes	Forward primer (5’-3’)	Reverse primer (5’-3’)	T°C
GPR178	AATGACCCTTTCTTCCCACTC	GTCTCTGTTCCTGTGTGCC	62
ACT; NM_031144	CACTGCCGCATCCTCTTCCT	AACCGCTCATTGCCGATAGTG	55
CYCLO; M19533	GAGCGTTTTGGGTCCAGGAAT	AATGCCCGCAAGTCAAAGAAA	55
HPRT;XM_34829	GGCACGAGGGACTTACCTCAC	GCGGGAAAAGCGGTCTGAG	55
GAPDH;X02231	ACATGCCGCCTGGAGAAACCT	GCCCAGGATGCCCTTTAGTGG	55
H3; NM_053985	ATTCGCAAGCTCCCCTTTCAG	TGGAAGCGCAGGTCTGTTTTGG	55
RPL19; NM_031103	TCGCCAATGCCAACTCTCGTC	AGCCCGGGAATGGACAGTCAC	55
TUB;NM_173102	CGGAAGGAGGCGGAGAGC	AGGGTGCCCATGCCAGAGC	55

(ACT) Beta-actin; (CYCLO) cyclophilin; (GAPDH) glyeraldehyde-3-phosphate-dehydrogenase; (H3) histone. (HPRT) hypoxanthine phosphoribosyl-transferase; RPL19 ribosomal protein L19 and (TUB) beta-tubulin beta 5.

### *In situ* hybridization

Three male C57BL/6J mice, ten weeks old, were anesthetized with an i.p injection of ketamine (7μg/kg bw) and medetomidine hydrochloride (70μg/kg bw) and transcardially perfused with saline followed 4% PFA/PBS. The brain was removed and postfixed in 4% PFA/PBS at 4°C overnight, embedded in 4% agarose and sectioned (70 μm) on a Vibratome (Leica, Sweden). Synthesis of cDNA probes and *in situ* hybridization were performed as previously described [15].

### Statistical analysis

Statistical analyses were performed using GraphPad Prism ver. 4 (San Diego, CA, USA). Results are expressed as mean ± SEM and considered statistically significant when p<0.05.Data were analysed by two-way ANOVA followed by post hoc LSD.

## Results

### Phylogenetic analysis

We retrieved amino acid sequences of GPR178 for 16 different species and HMM searches revealed only one copy of GPR178 in each species ranging from mammals to insects. GPR178 TM region does not share significant similarity with any other GPCR in any of these genomes, although its N-terminus revealed a structure similar to the Wnt domain of Frizzled family. Amino acid alignment showed considerable homology with the human orthologue, 91% with mouse and 93% with rat respectively (Fig 1). We also found sequences similar to GPR178 in the genomes from *Trichoplax adherens* and *Nematostella vectensis*, *(data not shown)*.

### Gene expression patterns in RT-PCR tissue panels

**Fig 3:**
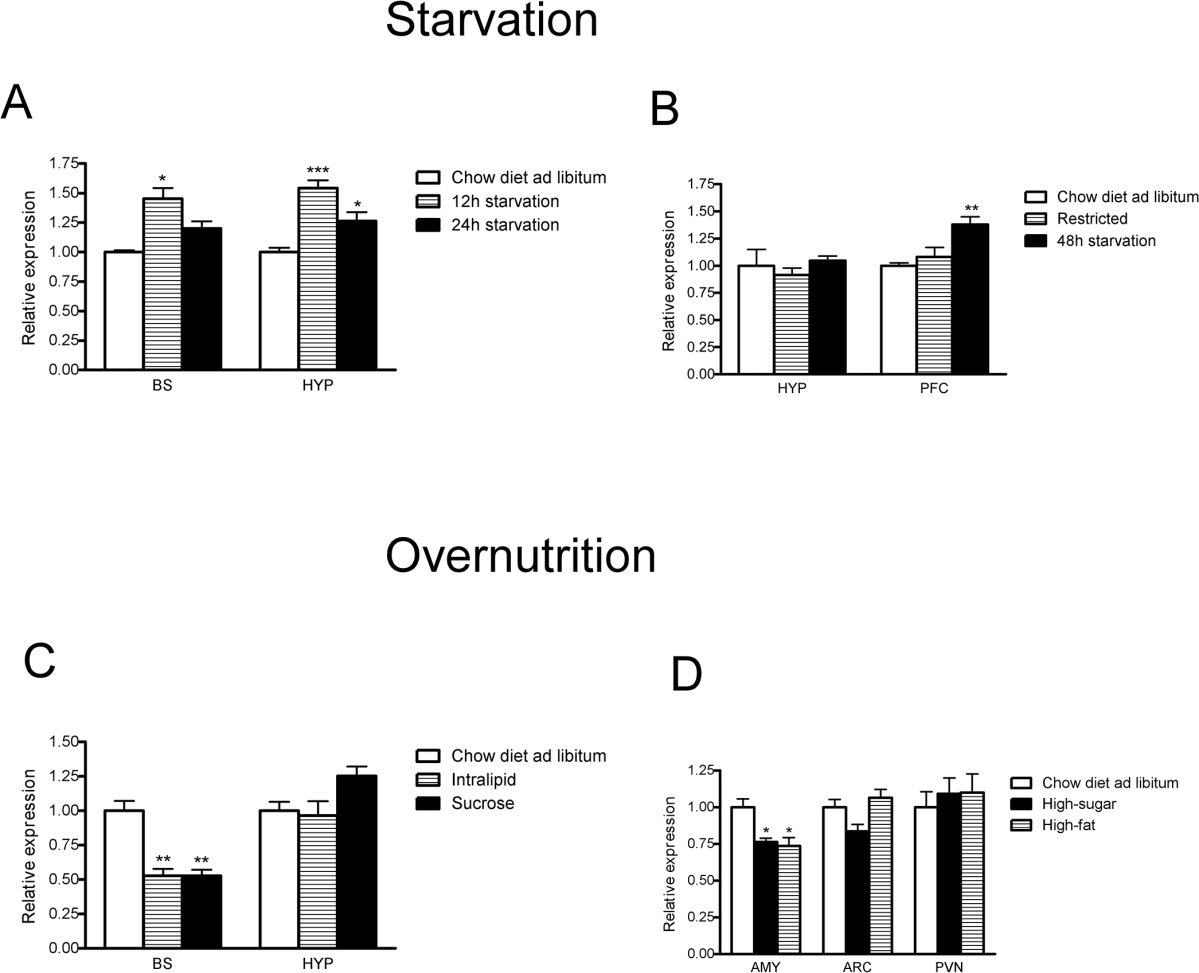
Gpr178 mRNA expression during food intake experiments. (A) Gpr178 mRNA expression after short-term exposure to starvation in mice; control group, had unlimited access to chow diet; n = 8 each group. (B) Gpr178 mRNA expression after long-term exposure (48 hours) in rat. Control group had unlimited access to chow diet, whereas restricted group was provided with 45% of the total daily caloric intake of the control group. Restricted group had free access to food until 48h before the end point of the experiment. N = 8 each group. (C) Gpr178 mRNA expression in mice after short-term (48 hours) full access to palatable drinking solutions (4.1% Intralipid or 10% sucrose); n = 8 each group. (D) Gpr178 mRNA expression in mice after long-term (10days) exposure to high-fat or high-carbohydrate diet; n = 8. BS, Brainstem; HYP, Hypothalamus; PFC, Prefrontal cortex; AMY, Amygdala. Data were analysed by two-way ANOVA followed by post hoc LSD (A, 12h starvation/24h starvation; B, restricted/48h starvation; C, Intralipid/10% sucrose; D, high-sugar/high-fat or high-carbohydrate diet). * p<0.05 ** p<0.01 ***p<0.001.

Gene expression levels of Gpr178 mRNA were measured in tissues isolated from the rat and mouse (Fig 2). In total, 32 and 21 tissues (rat and mouse, respectively) were included in our panels. Overall, Gpr178 mRNA was highly expressed in the rat CNS and the highest expression was detected in cross section V, 11.9 ± 0.8 and cross section VII, 9.3 ± 0.4. Moderate levels of Gpr178 expression were observed in cross section II (5.7 ± 0.2), cross section VI (7.4 ± 0,3), cross section VIII (5.3 ± 0.6), whereas similar levels were found in the brainstem (4.5 ± 0.1), cerebellum (5.6± 0.6), hypothalamus (4.8 ± 0.7), hippocampus (5.5 ± 0.7), eye (4.3 ± 0.4), intestine (6.3 ± 0.3), and olfactory bulb (4.9 ± 0.1). In the mouse, Gpr178 mRNA is highly expressed in the cerebellum (78.3 ± 15.1), lung (56.5 ± 19.2), whereas similar levels were found in the neocortex, striatum, hippocampus, brainstem and substantia nigra. Moderate to low Gpr178 mRNA levels were found in additional tissue types (Fig 2). Several diet paradigms were used to investigate Gpr178 mRNA levels in mice (detailed description in S1 Dataset). In an initial experiment, mice were exposed to both 12 and 24 hours of acute starvation. Our results indicated a significant hypothalamic and brainstem increase after 12 hours of starvation (p<0.05 and p<0.001, respectively). While mRNA transcription levels returned to control levels after 24 hours of starvation in the brainstem, this increase remained significant in the hypothalamus (Fig 3A). In line with these results, rats exposed to food restriction followed by long-term starvation (48 hours) showed significantly higher levels of Gpr178 mRNA in the prefrontal cortex compared to control group (p< 0.01). In the same experiment, Gpr178 mRNA expression remained unchanged in the hypothalamus and amygdala (Fig 3B). We evaluated the relationship between Gpr178 mRNA expression and short- and long-term overconsumption of palatable diets. Mice having short-term (48 hours) full access to palatable drinking solutions (4.1% Intralipid or 10% sucrose) had lower mRNA levels of Gpr178 in the brainstem compared to controls, while their hypothalamic levels remained unchanged (Fig 3C). No change in mRNA levels was observed after long-term (21 days) free access to the sugar solution, similarly to rats exposed to same treatment for 14 days (Data not shown). On the other hand, a decrease in the relative Gpr178 mRNA content was detected in the amygdala of rats fed the high-fat or high-carbohydrate diets (Fig 3D). No differences in expression levels of Gpr178 in the hypothalamus were observed.

### *In situ* hybridization (ISH)

**Fig 4:**
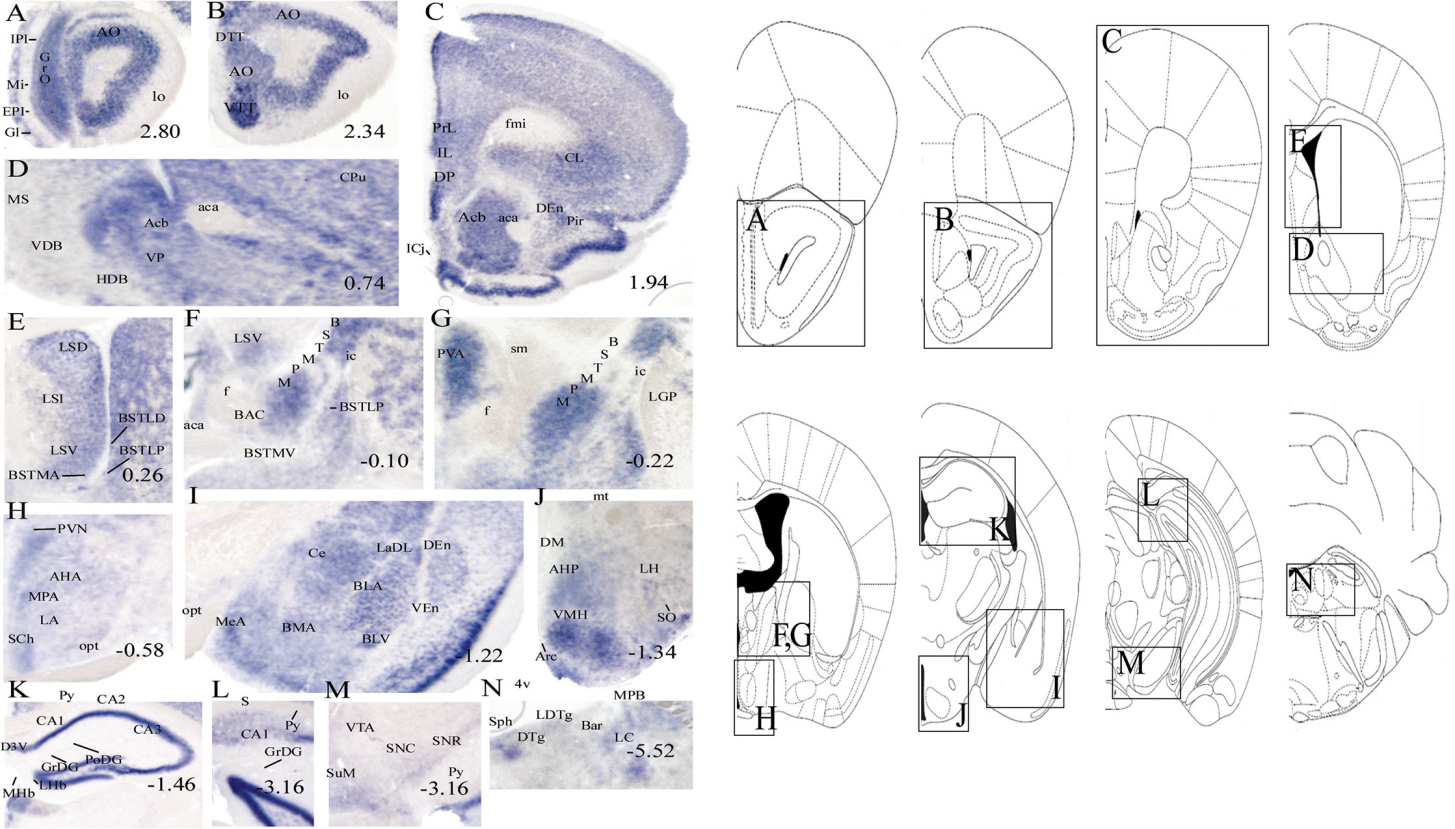
Floating *in situ* hybridization experiment in mouse brain. Floating *in situ* hybridization using 300 ng of digoxigenin labeled mouse Gpr178 antisense probe (A-N). Abbreviations and organization of the brain are depicted using Franklin and Paxinos [16].

ISH results in coronal sections from the mouse brain revealed consistency with our RT-PCR findings (Fig 4). Higher Gpr178 levels were detected in the amygdala, including the lateral amygdaloid nucleus (La) and the anterior part of the basolateral/basomedial amygdaloid nucleus (BLA/BMA) (Fig 4). Substantial expression was found in the olfactory bulb, nucleus accumbens (Acb), bed nucleus of the stria terminalis (BST) and several hypothalamic nuclei, including arcuate (Arc), paraventricular (PVN), suprachiasmatic (SCh) and ventromedial nucleus (VMH). In addition, Gpr178 expression was abundant in the pyramidal and granular cell layer of the hippocampus (Table 3).

**Table 3:** CNS expression of Gpr178 in adult male C57BI/6 mouse.

Accumbens nucleus (Acb)	+++
Anterior hypothalamic area, anterior part (AHA)	0/+
Anterior hypothalamic area, posterior part (AHP)	+
Anterior olfactory nucleus (AO)	+++
Anterior pretectal nucleus (APT)	0
Arcuate hypothalamic nucleus (Arc)	++
Barrington´s nucleus (Bar)	0
Basolateral amygdaloid nucleus, anterior part (BLA)	++/++
Basolateral amygdaloid nucleus, ventral part (BLV)	0
Basomedial amygdaloid nucleus, anterior part (BMA)	+++
Bed nucleus of stria terminalis, supracapsular part (BSTS)	+
Bed nucleus of the anterior commissure (BAC)	++
Bed nucleus of the stria terminalis, lateral division, dorsal part (BSTLD)	++
Bed nucleus of the stria terminalis, lateral division, posterior part (BSTLP)	++
Bed nucleus of the stria terminalis, medial division, anterior part (BSTMA)	+
Bed nucleus of the stria terminalis, medial division, posterolateral part (BSTMPM)	++
Bed nucleus of the stria terminalis, medial division, ventral part (BSTMV)	++
CA1 of hippocampus (CA1)	0
CA2 of hippocampus (CA2)	0
CA3 of hippocampus (CA3)	0
Caudate putamen (striatum) (CPu)	++
Central amygdaloid nucleus (Ce)	+++
Centrolateral thalamic nucleus (CL)	++/+++
Dorsal endopiriform nucleus (DEn)	+/++
Dorsal peduncular cortex (DP)	+++
Dorsal tegmental nucleus (DTg)	0
Dorsal tenia tecta (DTT)	+++
Dorsomedial hypothalamic nucleus (DM)	+
External plexiform layer of the olfactory bulb (EPl)	0
Glomerular layer of the olfactory bulb (Gl)	++
Granular cell layer of the olfactory bulb (GrO)	+++
Granular layer of the dentate gyrus (GrDG)	+++
Infralimbic cortex (IL)	++
Internal plexiform layer of the olfactory bulb (IPl)	0
Island of Calleja (ICj)	+++
Lateral amygdaloid nucleus, dorsolateral part (LaDL)	+++
Lateral amygdaloid nucleus, ventrolateral part (LaVL)	+++
Lateral globus pallidus (LGP)	0
Lateral habenular nucleus (LHb)	++
Lateral hypothalamic area (LH)	+
Lateral olfactory tract (lo)	0
Lateral preoptic area (LPO)	0
Lateral septal nucleus, dorsal part (LSD)	++/++
Lateral septal nucleus, intermediate part (LSI)	+
Lateral septal nucleus, ventral part (LSV)	++/++
Laterodorsal tegmental nucleus (LDTg)	0
Lateroanterior hypothalamic nucleus (LA)	0/+
Locus coeruleus (LC)	+/++
Magnocellular preoptic nucleus (MCPO)	0
Medial amygdaloid nucleus, anterior part (MeA)	+++
Medial amygdaloid nucleus, posterodorsal part (MePD)	+++
Medial amygdaloid nucleus, posteroventral part (MePV)	+++
Medial geniculate nucleus (MG)	0
Medial habenular nucleus (MHb)	+++
Medial mammillary nucleus, medial part (MM)	0
Medial parabrachial nucleus (MPB)	+
Medial preoptic area (MPA)	0/+
Medial preoptic nucleus (MPO)	+
Medial septal nucleus (MS)	0
Median preoptic nucleus (MnPO)	++
Mitral cell layer of the olfactory bulb (Mi)	+++
Nucleus of the horizontal limb of the diagonal band (HDB)	0
Nucleus of the vertical limb of the diagonal band (VDB)	0
Paraventricular thalamic nucleus (PVN)	++
Paraventricular thalamic nucleus, anterior part (PVA)	++
Periaqueductal gray (PAG)	0
Piriform cortex (Pir)	0/+
Polymorph layer of the dentate gyrus (PoDG)	+
Posterior intralaminar thalamic nucleus (PIL)	+
Posterior pretectal nucleus (PPT)	0
Prelimbic cortex (PrL)	+++
Pyramidal cell layer of the hippocampus (Py)	+++
Reticular thalamic nucleus (Rt)	0
Reuniens thalamic nucleus (Re)	0
Septofimbrial nucleus (SFi)	0
Septohippocampal nucleus (SHi)	0/+
Sphenoid nucleus (Sph)	++
Subiculum (S)	++
Substantia innominata (SI)	0
Substantia nigra, compact part (SNC)	+
Substantia nigra, reticular part (SNR)	0
Suprachiasmatic nucleus (SCh)	+/++
Supramammillary nucleus (SuM)	+
Supraoptic nucleus (SO)	+
Ventral endopiriform nucleus (VEn)	0/+
Ventral pallidum (VP)	+++
Ventral tegmental area (VTA)	0
Ventral tenia tecta (VTT)	+++
Ventromedial hypothalamic nucleus (VMH)	++
Zona incerta (ZI)	0

Expression values are obtained from manual inspection of in situ hybridized mouse coronal brain sections[17]. Brain regions are organized in alphabetical order. Scale: 0, not detectable; + low expression; ++, medium expression;+++, high expression.

## Discussion

GPR178 is an orphan receptor whose biological function is still unknown. For the first time, this study clarifies the evolutionary history of this receptor, its tissue distribution in rat and mouse, and demonstrates that the expression levels of Gpr178 mRNA are altered only during acute starvation and exposure to a high-fat and high-sugar diets, with no changes during long-term exposure to palatable diet.

Our phylogenetic evaluation indicates that GPR178 is highly conserved among vertebrates and insects (Fig 1). GPR178 from all vertebrates included in this study has at least 70% amino acid identify with human GPR178. This is significantly higher than for most other GPCRs, for example, the mean amino acid identity between human and chicken GPCRs is 72.9% [18], while chicken GPR178 shares 84% amino acid identity with human GPR178. Another interesting observation is that there are no other genes in any of the species investigated with high similarity to GPR178 (Table 1). The high degree of conservation between GPR178 orthologs could be due to the fact that GPR178, unlike most other GPCRs, is not part of a receptor family with several members sharing the same ligand(s) and having similar physiological functions, resulting in a high pressure for evolutionary conservation. Interestingly, we also identify a putative ortholog to GPR178 in *Trichoplax adherens* which is considered one of the earliest branches in animal evolution. This suggests that GPR178 was present at or before the origin of animals. Taken together this would implicate a conserved function of GPR178, at least at the cellular level, including possible ligand interaction.

We profiled Gpr178 mRNA expression in a panel of rat and mouse tissues. Gpr178 gene expression was detected in both central and peripheral tissues, including several organs, revealing a species-specific distribution of the gene, suggesting a difference in physiological function in mice and rats (Fig 2). In the rat, the highest mRNA levels were found in the brainstem, hypothalamus and intestine, with moderate levels in the prefrontal cortex and in the eye, whereas in the mouse, the highest expression was detected in the brain (neocortex, hypothalamus, striatum) and lung. A detailed murine brain distribution of the gene was studied using *in situ* hybridization (ISH), confirming that Gpr178 has high CNS expression (Fig 4). ISH results were in good agreement with the PCR results in mice. High Gpr178 expression was found in hypothalamic nuclei, including the arcuate (ARC), ventromedial (VMH) and paraventricular (PVN) nuclei, recognized as critical sites integrating central and peripheral information involved in appetite regulation [19, 20] (Table 3). Similarly, high intensity was observed in areas in the nucleus accumbens and amygdala, physiological relevant areas implicated in motivation for food and reward [21, 22] and this lead us to speculate that the gene could be a candidate for food intake signalling in the limbic system [23, 24]. In order to further investigate this hypothesis, we examined the relationship between changes in food intake and the variation of Gpr178 mRNA expression in a series of experiments. Our results showed that Gpr178 mRNA expression in the hypothalamus and the brainstem is altered following acute energy changes of both rat and mouse.

In an initial experiment, we examined Gpr178 mRNA expression in the hypothalamus and brainstem of food-deprived versus *ad libitum-*fed mice (Fig 3A). Our results revealed that acutely starved mice showed increased Gpr178 transcript levels in both tissues examined. In the brainstem, transcription levels were similar to the control group after 24 h of starvation, whereas in the hypothalamus upregulation was still detected. A large number of neural mediators involved in the regulation of food intake are produced in hypothalamic various nuclei, including the ARC, PVN, VMH, DMH and LH [20]. Our ISH analysis detected relatively high Gpr178 mRNA level in VMH, known to be involved in satiety mechanisms [25]. Marked Gpr178 mRNA levels were found in the ARC, PVN and LH. Brain lesion and stimulation studies have demonstrated that neurons in the ARC projects to LH “the hunger centre” and to VMH “the satiety centre” [26] identifying a bi-directional neuronal traffic [20]. On the other hand, chronic food restriction upregulated Gpr178 transcription only in the rat PFC, an area of the brain involved in the control of food intake [27, 28] (Fig 3B). It has been recently demonstrated that the activity of D1-type dopamine receptor–expressing neurons (D1) in the medial PFC is enhanced during feeding, whereas their inhibition has opposite effects [28]. The downstream target of D1 neurons are found in the medial basolateral amygdala, and in our study, ISH showed a dense occurrence of Gpr178 mRNA in the amygdaloid nuclei. Gpr178 content in the amygdala after chronic food restriction showed no difference compared to control animals and our results might suggest a new a plausible implication of Gpr178 in the circuit food intake control via D1 neurons in PFC and amygdala.

To further investigate changes in Gpr178 expression following high palatable food consumption, rats were fed either high-fat diet or high-sugar diet (Fig 3C). After 48 hours of unrestricted diet, Gpr178 expression was decreased in the brainstem, whereas hypothalamic levels remained unchanged excluding hypothalamic involvement of the gene in the long term control of feeding mechanisms. Surprisingly, in another diet paradigm assessing the receptor expression after long-term exposure to palatable food, we observed that Gpr178 mRNA levels were significantly downregulated in amygdala following high-fat and high-sugar consumption. In the same experiment, unchanged transcript levels were found after chow diet consumption suggesting a plausible implication of the receptor in the complex circuitry of hedonic feeding via reward mechanisms [29].

As GPCRs mediates several physiological responses, their expression levels can be highly responsive to environmental factors and contingent on the cellular context [30]. Hence, further studies are needed to elucidate the role of Gpr178 expression in food intake, as GPCRs transcription levels might change in relation to the activity of their ligands such as hormones and sensorial stimuli [31]. Taken together, our results demonstrated that transcription levels of Gpr178 are highly susceptible to acute exposure to starvation or acute exposure to palatable food. No implication of involvement of Gpr178 in response to long-term exposure to palatable diet was observed. Our results further suggest a role of Gpr178 in amygdala as functional regulator in the complex mechanisms of feeding reward circuitry.

## Supporting Information

S1 Dataset (PZF)

S2 Dataset (PZF)

## References

[1] Almen MS, Nordstrom KJ, Fredriksson R, Schioth HB. Mapping the human membrane proteome: a majority of the human membrane proteins can be classified according to function and evolutionary origin. BMC biology. 2009;7:50 Epub 2009/08/15. 10.1186/1741-7007-7-50 19678920 PMC2739160

[2] Lagerstrom MC, Schioth HB. Structural diversity of G protein-coupled receptors and significance for drug discovery. Nature reviews Drug discovery. 2008;7(4):339–57. Epub 2008/04/03. 10.1038/nrd2518 . 18382464

[3] Fredriksson R, Lagerstrom MC, Lundin LG, Schioth HB. The G-protein-coupled receptors in the human genome form five main families. Phylogenetic analysis, paralogon groups, and fingerprints. Molecular pharmacology. 2003;63(6):1256–72. Epub 2003/05/23. 10.1124/mol.63.6.1256 . 12761335

[4] Gloriam DE, Fredriksson R, Schioth HB. The G protein-coupled receptor subset of the rat genome. BMC genomics. 2007;8:338 Epub 2007/09/26. 10.1186/1471-2164-8-338 17892602 PMC2117022

[5] Brauner-Osborne H, Wellendorph P, Jensen AA. Structure, pharmacology and therapeutic prospects of family C G-protein coupled receptors. Current drug targets. 2007;8(1):169–84. Epub 2007/02/03. . 17266540 10.2174/138945007779315614

[6] Krasnoperov VG, Bittner MA, Beavis R, Kuang Y, Salnikow KV, Chepurny OG, et al alpha-Latrotoxin stimulates exocytosis by the interaction with a neuronal G-protein-coupled receptor. Neuron. 1997;18(6):925–37. Epub 1997/06/01. . 9208860 10.1016/s0896-6273(00)80332-3

[7] Nordstrom KJ, Fredriksson R, Schioth HB. The amphioxus (Branchiostoma floridae) genome contains a highly diversified set of G protein-coupled receptors. BMC Evol Biol. 2008;8:9 Epub 2008/01/18. 10.1186/1471-2148-8-9 18199322 PMC2246102

[8] Cadigan KM, Peifer M. Wnt signaling from development to disease: insights from model systems. Cold Spring Harbor perspectives in biology. 2009;1(2):a002881 Epub 2010/01/13. 10.1101/cshperspect.a002881 20066091 PMC2742092

[9] Civelli O, Reinscheid RK, Zhang Y, Wang Z, Fredriksson R, Schioth HB. G protein-coupled receptor deorphanizations. Annual review of pharmacology and toxicology. 2013;53:127–46. Epub 2012/10/02. 10.1146/annurev-pharmtox-010611-134548 . 23020293 PMC5828024

[10] Altschul SF, Wootton JC, Gertz EM, Agarwala R, Morgulis A, Schaffer AA, et al Protein database searches using compositionally adjusted substitution matrices. The FEBS journal. 2005;272(20):5101–9. Epub 2005/10/13. 10.1111/j.1742-4658.2005.04945.x 16218944 PMC1343503

[11] Bateman A, Birney E, Durbin R, Eddy SR, Finn RD, Sonnhammer EL. Pfam 3.1: 1313 multiple alignments and profile HMMs match the majority of proteins. Nucleic acids research. 1999;27(1):260–2. Epub 1998/12/10. 9847196 10.1093/nar/27.1.260 PMC148151

[12] Jones DT, Taylor WR, Thornton JM. The rapid generation of mutation data matrices from protein sequences. Computer applications in the biosciences: CABIOS. 1992;8(3):275–82. Epub 1992/06/01. . 1633570 10.1093/bioinformatics/8.3.275

[13] Lagerström MC, Rabe N, Haitina T, Kalnina I, Hellström AR, Klovins J, et al The evolutionary history and tissue mapping of GPR123: specific CNS expression pattern predominantly in thalamic nuclei and regions containing large pyramidal cells. Journal of Neurochemistry. 2007;100(4):1129–42. 10.1111/j.1471-4159.2006.04281.x 17212699

[14] Vandesompele J, De Preter K, Pattyn F, Poppe B, Van Roy N, De Paepe A, et al Accurate normalization of real-time quantitative RT-PCR data by geometric averaging of multiple internal control genes. Genome biology. 2002;3(7):RESEARCH0034 Epub 2002/08/20. 12184808 10.1186/gb-2002-3-7-research0034 PMC126239

[15] Lagerstrom MC, Rabe N, Haitina T, Kalnina I, Hellstrom AR, Klovins J, et al The evolutionary history and tissue mapping of GPR123: specific CNS expression pattern predominantly in thalamic nuclei and regions containing large pyramidal cells. J Neurochem. 2007;100(4):1129–42. . 17212699 10.1111/j.1471-4159.2006.04281.x

[16] Paxinos G, Franklin KBJ. Paxinos and Franklin's the Mouse Brain in Stereotaxic Coordinates. 3 ed. New York: Academic Press; 2007.

[17] Sundberg BE, Waag E, Jacobsson JA, Stephansson O, Rumaks J, Svirskis S, et al The evolutionary history and tissue mapping of amino acid transporters belonging to solute carrier families SLC32, SLC36, and SLC38. Journal of molecular neuroscience: MN. 2008;35(2):179–93. 10.1007/s12031-008-9046-x . 18418736

[18] Lagerstrom MC, Hellstrom AR, Gloriam DE, Larsson TP, Schioth HB, Fredriksson R. The G protein-coupled receptor subset of the chicken genome. PLoS Comput Biol. 2006;2(6):e54 Epub 2006/06/03. 10.1371/journal.pcbi.0020054 16741557 PMC1472694

[19] Yeo GS, Heisler LK. Unraveling the brain regulation of appetite: lessons from genetics. Nature neuroscience. 2012;15(10):1343–9. Epub 2012/09/26. 10.1038/nn.3211 . 23007189

[20] Schwartz MW, Woods SC, Porte D Jr, Seeley RJ, Baskin DG. Central nervous system control of food intake. Nature. 2000;404(6778):661–71. Epub 2000/04/15. 10.1038/35007534 . 10766253

[21] Pecina S, Cagniard B, Berridge KC, Aldridge JW, Zhuang X. Hyperdopaminergic mutant mice have higher "wanting" but not "liking" for sweet rewards. The Journal of neuroscience: the official journal of the Society for Neuroscience. 2003;23(28):9395–402. Epub 2003/10/17. . 14561867 10.1523/JNEUROSCI.23-28-09395.2003 PMC6740586

[22] Mietlicki-Baase EG, Ortinski PI, Rupprecht LE, Olivos DR, Alhadeff AL, Pierce RC, et al. The food intake-suppressive effects of glucagon-like peptide-1 receptor signaling in the ventral tegmental area are mediated by AMPA/kainate receptors. American journal of physiology Endocrinology and metabolism. 2013. Epub 2013/10/10. 10.1152/ajpendo.00413.2013 . 24105414 PMC3882373

[23] Ahn S, Phillips AG. Modulation by central and basolateral amygdalar nuclei of dopaminergic correlates of feeding to satiety in the rat nucleus accumbens and medial prefrontal cortex. The Journal of neuroscience: the official journal of the Society for Neuroscience. 2002;22(24):10958–65. Epub 2002/12/18. . 12486191 10.1523/JNEUROSCI.22-24-10958.2002 PMC6758436

[24] Carlini VP, Varas MM, Cragnolini AB, Schioth HB, Scimonelli TN, de Barioglio SR. Differential role of the hippocampus, amygdala, and dorsal raphe nucleus in regulating feeding, memory, and anxiety-like behavioral responses to ghrelin. Biochemical and biophysical research communications. 2004;313(3):635–41. Epub 2003/12/31. . 14697239 10.1016/j.bbrc.2003.11.150

[25] Satoh N, Ogawa Y, Katsuura G, Tsuji T, Masuzaki H, Hiraoka J, et al Pathophysiological Significance of the Obese Gene Product, Leptin, in Ventromedial Hypothalamus (VMH)-Lesioned Rats: Evidence for Loss of Its Satiety Effect in VMH-Lesioned Rats. Endocrinology. 1997;138(3):947–54. 10.1210/endo.138.3.4989 . 9048594

[26] Stellar E. The physiology of motivation. 1954. Psychological review. 1994;101(2):301–11. Epub 1994/04/01. . 8022960 10.1037/0033-295x.101.2.301

[27] Davidson TL, Chan K, Jarrard LE, Kanoski SE, Clegg DJ, Benoit SC. Contributions of the hippocampus and medial prefrontal cortex to energy and body weight regulation. Hippocampus. 2009;19(3):235–52. Epub 2008/10/03. 10.1002/hipo.20499 18831000 PMC2649976

[28] Land BB, Narayanan NS, Liu RJ, Gianessi CA, Brayton CE, Grimaldi DM, et al Medial prefrontal D1 dopamine neurons control food intake. Nature neuroscience. 2014;17(2):248–53. Epub 2014/01/21. 10.1038/nn.3625 24441680 PMC3968853

[29] Zhang Q, Li H, Guo F. Amygdala, an important regulator for food intake. Front Biol. 2011;6(1):82–5. 10.1007/s11515-011-0950-z

[30] Bermak JC, Li M, Bullock C, Zhou QY. Regulation of transport of the dopamine D1 receptor by a new membrane-associated ER protein. Nat Cell Biol. 2001;3(5):492–8. Epub 2001/05/02. 10.1038/35074561 . 11331877

[31] Ritter SL, Hall RA. Fine-tuning of GPCR activity by receptor-interacting proteins. Nat Rev Mol Cell Biol. 2009;10(12):819–30. Epub 2009/11/26. 10.1038/nrm2803 19935667 PMC2825052

